# Bionomic aspects of dengue vectors *Aedes aegypti* and *Aedes albopictus* at domestic settings in urban, suburban and rural areas in Gampaha District, Western Province of Sri Lanka

**DOI:** 10.1186/s13071-022-05261-3

**Published:** 2022-04-27

**Authors:** Rasika Dalpadado, Deepika Amarasinghe, Nayana Gunathilaka, Nalin Ariyarathna

**Affiliations:** 1 Regional Director of Health Services Office, Gampaha District, Gampaha, Sri Lanka; 2 Department of Zoology and Environmental Management, Faculty of Science, University of Kelaniya, Dalugama, Sri Lanka; 3 Department of Parasitology, Faculty of Medicine, University of Kelaniya, Ragama, Sri Lanka

**Keywords:** *Aedes aegypti*, *Aedes albopictus*, Gampaha, Resting sites, IRS, Dengue

## Abstract

**Background:**

The lack of information on behavioural patterns of *Aedes aegypti* and *Aedes albopictus* has become a significant limitation in vector control and disease management programmes. Therefore, the current study was focused on determining some bionomics aspects: breeding, resting, host-seeking and feeding preferences of *Ae. aegypti* and *Ae. albopictus* in Sri Lanka.

**Methods:**

Larval and adult surveys were conducted from April 2017 to April 2019 monthly in six selected Medical Officer of Health (MOH) areas in Gampaha Distinct, Western province, Sri Lanka, representing urban, suburban and rural settings. Resting preferences of adult mosquitoes were observed from indoor and outdoor places using a Prockopack aspirator. The information on resting height, surface, material and locality was recorded. Human-baited double-net traps were used to determine the host-seeking time of *Aedes* mosquitoes. Statistical differences in the spatial distribution of mosquitoes in selected MOH areas and prevalence of vectors were analysed using general linear model (GLM). A chi-square test was used to analyse the resting behaviour.

**Results:**

Total of 19,835 potential breeding sites were examined at 13,563 premises, and 18.5% (*n* = 1856) were positive for *Aedes* larvae. Distribution of *Ae. aegypti* and *Ae. albopictus* was statistically significant at species level (*df* = 1; *F* = 137.134; *P* < 0.05 GLM) and study setting (*df* = 2; *F* = 8.125; *P* < 0.05). *Aedes aegypti* breeding was found mainly in temporary removals (18.8%; *n* = 34), discarded non-degradables (12.15%; *n* = 22) and tyres (9.95%; *n* = 18). Natural (14.7%; *n* = 246) and temporary removals (13.6%; *n* = 227) and discarded non-reusable items were the key ovipositing sites for *Ae. albopictus*. In the adult mosquito survey, the majority was comprised of *Ae. albopictus* (54.5%; *n* = 999), which denoted exophilic nature (90.8%; *n* = 758), and 45.5% (*n* = 835) represented by *Ae. aegypti* mosquitoes who were mainly endophilic (84.3%; *n* = 842). *Aedes aegypti* rested on cloth hangings and curtains, followed by the furniture, while *Aedes albopictus* was predominant in outdoor vegetation. In both vectors, biting patterns denoted a typical diurnal pattern with two peaks of host-seeking and biting activity in the morning and afternoon.

**Conclusions:**

The majority (80%) of the larval habitats were artificial containers. The use of larvicides for vector control as the prominent measure is questionable since applying these chemicals may target only 20% of the total breeding grounds, which are permanent. The resting places of adult mosquitoes are mainly indoors. Therefore, using thermal space spraying of insecticide may not be appropriate, and indoor residual spraying is recommended as a suitable intervention to target adult mosquitoes. This study warrants a holistic vector control approach for all medically important mosquitoes and insects, ensuring the rational use of finance and resources.

**Graphical Abstract:**

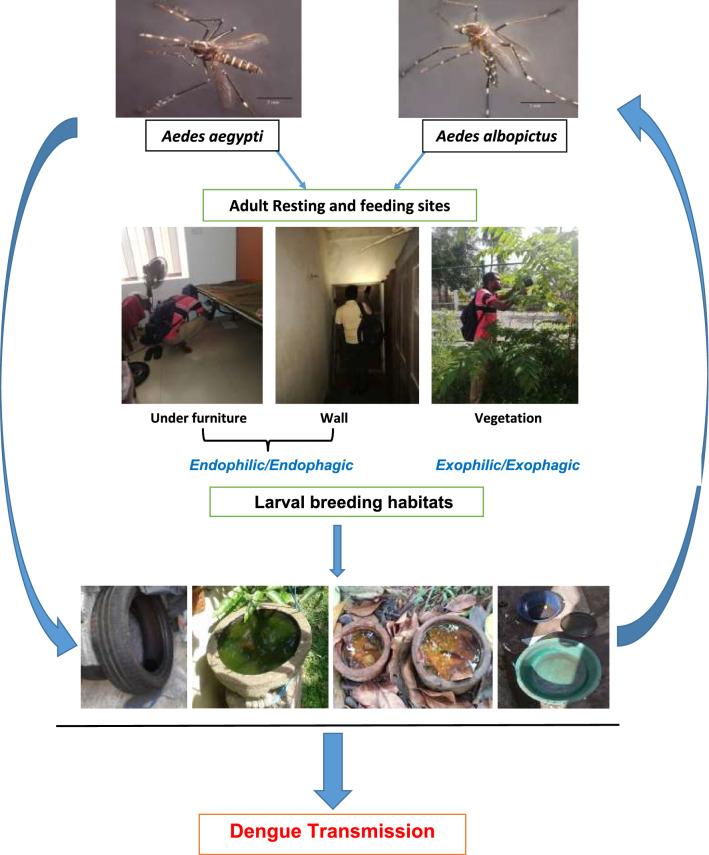

**Supplementary Information:**

The online version contains supplementary material available at 10.1186/s13071-022-05261-3.

## Background

Some species of the Genus *Aedes* are medically important for transmitting arboviruses which cause dengue, chikungunya, yellow fever and Zika to humans [1–4]. Of the recorded species, *Aedes aegypti* and *Aedes albopictus* received immediate attention since these two species have been identified as vectors of dengue transmission [5]. According to the records, *Aedes aegypti* originated in Africa as tree-hole forest mosquitoes [6]. In contrast, the Asian tiger mosquito *Aedes albopictus* is native to tropical and subtropical areas of Southeast Asia and breeds in natural habitats, including tree holes, bamboo stumps and bromeliads at the edges of tropical forests [7]. However, with time they spread to other regions worldwide through travel and trade [8] and reformed to breed in human-made containers/micro-breeding habitats in urban setup [6–8].

In the Southeast Asian region, *Ae. aegypti* is considered as the principal vector of dengue. *Aedes albopictus* has been recognized as the secondary vector of the dengue, which is also important in the maintenance of the virus [9]. Furthermore, *Ae. albopictus* has become one of the most invasive species globally because of its strong ability to adapt to new environments [7, 10]. These mosquitoes can be infected by the virus during their feeding process and, once infected, the mosquito can retain the virus throughout its adult life [11].

In the case of insect-borne disease transmission, biology, bionomics and the life history of vectors are important aspects that could influence the efficiency to transmit diseases. There can be changes in the biology and bionomics of vector species in different regions. Furthermore, the availability and composition of vectors may also vary with different spatial setups. *Aedes aegypti* is a strongly anthropophilic mosquito adapted to live around humans at a domestic setup. Therefore, the mosquito is more predominant in urban settings than in rural areas as its abundance is positively correlated with increasing urbanization. It is considered the most efficient vector of the dengue virus even at low densities [12, 13]. In some countries, *Ae. albopictus* leads to an outbreak situation of dengue incidence. The outbreak of dengue and chikungunya in Madagascar (Toamasina) during 2006 showed that *Ae. albopictus* is the only urban vector [14]. It is evidenced that the biology and behavioural aspects of dengue vectors vary in different regions of the globe [5].

Unplanned urbanization, globalization of the world with travel and trade, human population growth and suitable climatic conditions directly correlates with the expansion of dengue vector distribution and dengue transmission, especially in low- and middle-income countries in tropical and subtropical regions [15–17]. Therefore, understanding the prevalence of vector species, their behaviours and important bionomic aspects at different urban, suburban and rural settings would provide more practical and reliable information to develop more effective, precise and focused vector control strategies. Resting and feeding behaviours of vectors are important facts since they are prerequisites to determine their role in disease transmission in endemic settings [17]. The behavioural and physiological processes that may account for the presence of resting behaviour of *Ae. aegypti* inside houses and their implications for dengue outbreak interventions have been revealed [18].

In Sri Lanka, insecticidal space-spraying is extensively used as a routine dengue control activity [19]. The efficacy of this method depends on targeted mosquito species, their susceptibility to insecticides, indoor penetration capacity of the insecticides, frequency/timing of applications and targeting of appropriate sites [20]. Most importantly, the application of space-spraying also should be related to the behaviour of the targeted species [20]. However, investigations on such behavioural, biological and bionomic aspects of dengue vectors in Sri Lanka are scarce. This has become a significant limitation in vector control and disease management programmes. Therefore, this study aimed to investigate the biology, bionomics and behavioural aspects of dengue vectors in rural, suburban and urban areas in the Gampaha District; the western province of Sri Lanka contributes the second-highest number of cases of dengue in the country.

## Methods

### Study area

**Fig. 1:**
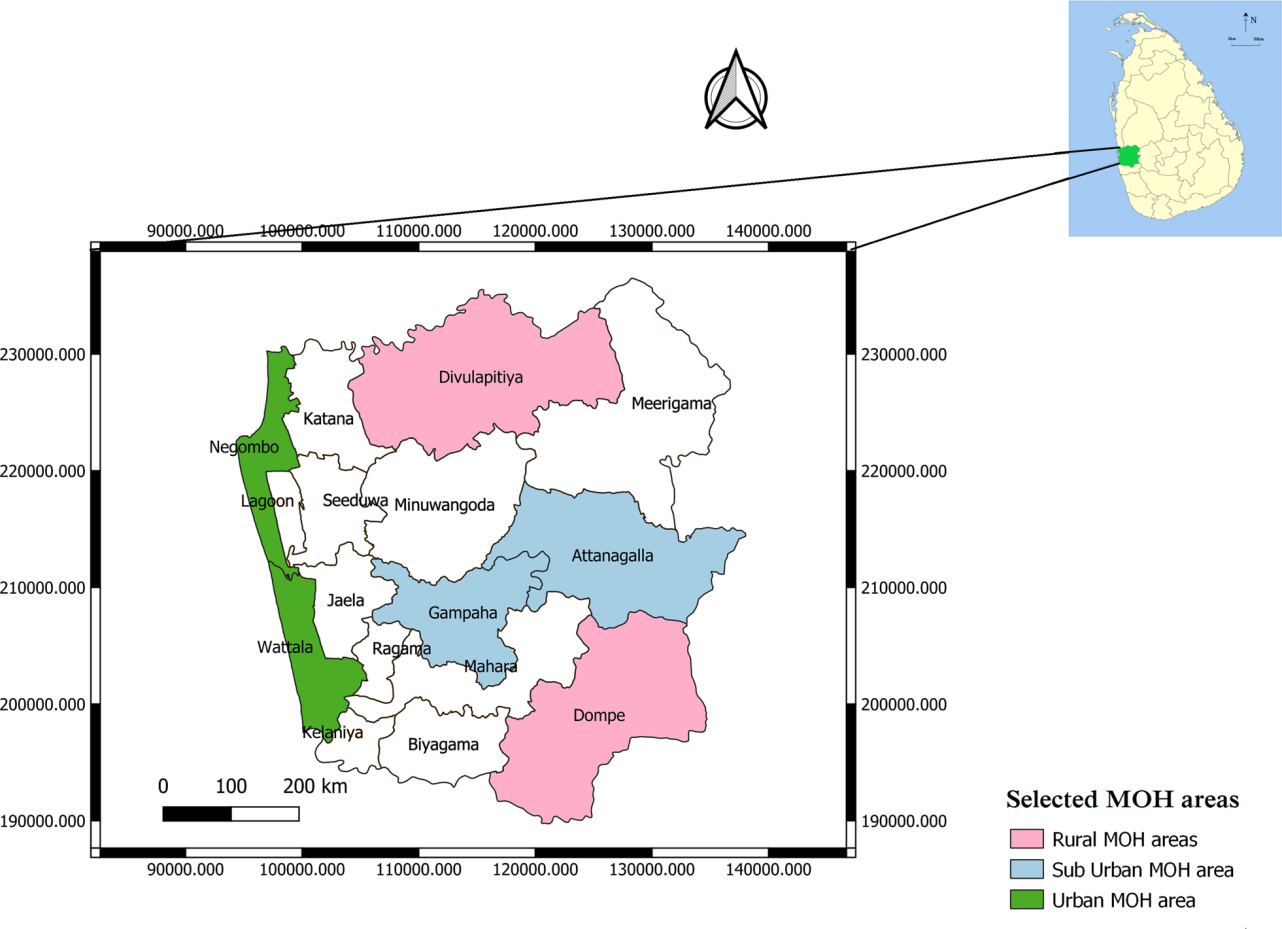
Map showing MOH areas in the Gampaha District, Sri Lanka, highlighting the selected MOH areas representing urban, suburban and rural environmental settings

Gampaha district of Sri Lanka covers an area of 1387 km^2^. It has a human population of 2,574,324, recorded as the highest residential population in Sri Lanka. The annual rainfall is about 2500 mm, mainly during two monsoonal periods from April to June and October to December [21]. The District of Gampaha comprises 16 Medical Officer of Health (MOH) areas. The present study was conducted in six selected Medical Officer of Health (MOH) areas (Fig. 1), covering an estimated human population of 1.2 million. In selecting sites, areas with different environmental settings (urban, suburban and rural) were selected randomly. Geographic areas located inside towns and cities were described as urban. In contrast, rural areas are located outside towns and cities and are usually less developed with significant land cover under agriculture and natural vegetation. Areas with mixed characteristics were considered suburban [12]. Accordingly, Negombo (population density per km^2^: 4292/km^2^) and Wattala (4191/km^2^) MOH areas were identified as urban and Attanagalla (1447/km^2^) and Gampaha (2357/km^2^) MOH areas as suburban. Dompe (1052/km^2^) and Divulapitiya (836/km^2^) MOH areas represent rural setups.

### Entomological investigations and bionomics aspects of *Aedes* mosquitoes

Entomological surveys for both larval and adult stages of *Aedes* mosquitoes and the biology/bionomics aspects were conducted from April 2017 to December 2019 monthly using standard entomological techniques according to the guidelines specified by the World Health Organization and National Dengue Control Unit of Sri Lanka [22, 23]. At each sampling attempt, a locality was selected, considering a central point for entomological surveillance. The survey was conducted within 200–300 m at each selected locality.

### Collection of mosquito larvae from breeding habitats

The larval collections were performed, covering all potential permanent and temporary breeding sites using standard dipping, siphoning and pipetting methods depending on the nature in the breeding habitat [24]. All positive and potential breeding sites were recorded and categorized under 20 different types including water storage containers (water storage barrels, water storage cement tanks, water storage other containers), concrete slabs, gutters, tyres, ornamentals (flower vases, fish tanks), natural breeding places (leaf axils, tree holes, bamboo stumps), ponds, shallow wells, tube wells, A/C and refrigerator trays, temporary removal items (household utensils, machinery, machinery parts stored in backyard/outside of premises without shade for future usage), covering items/polythene, discarded degradable (damaged clay pots, coconut shells), discarded non-reusable items (damaged ceramic items, tin, can, non-reusable plastic containers), discarded reusable items (glass bottles, tyres, reusable plastic containers), pet feeding cups, non-used commodes and cisterns. All other larval habitats were classified as other.

Field-collected larvae were classified into early (1st and 2nd) and late (3rd and 4th) instars and transferred into transparent plastic vials (5 ml) using a pasture pipette. The larvae were transferred safely to the laboratory. Larvae of 3rd and 4th instars were directly taken for species identification using morphological taxonomic keys [25]. Early instars were reared under optimized laboratory conditions with larval food until 3rd instar stage to confirm the species identification. The prevalence of mosquitoes in different breeding habitat categories was calculated as a percentage of containers positive for each species out of the total number of positive containers.

### Adult mosquito collection from resting places

**Fig. 2:**
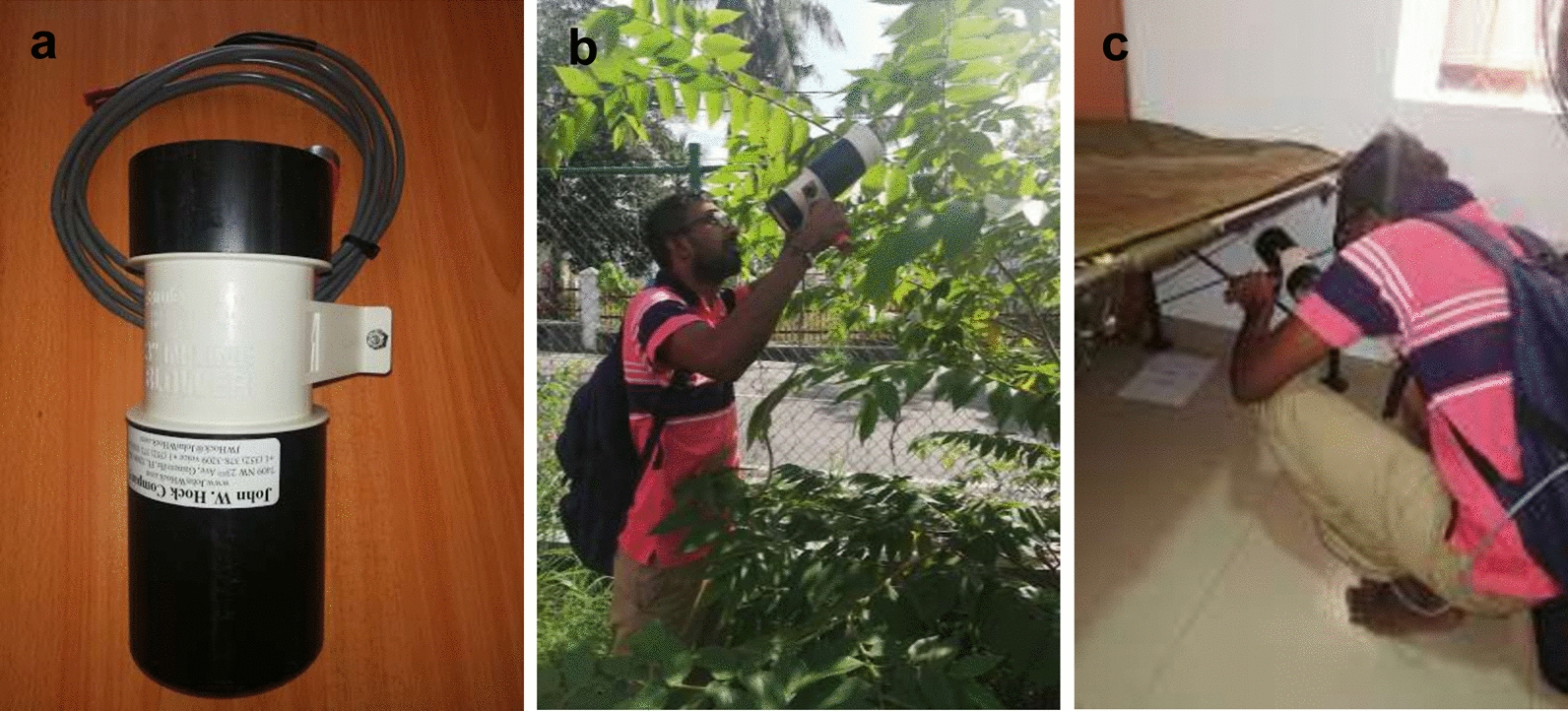
Field collection of adult mosquitoes using Prokopack aspiration. **a** Prokopack aspirator (model: 140) used for surveys. **b** Outdoor collection of mosquitoes, **c** indoor collection of mosquitoes

Adult mosquitoes were collected in both outdoor and indoor places of 15 randomly selected premises once in 2 months in each selected MOH area using a Prokopack aspirator (John W. Hock Co., Gainesville, FL, USA, Model: 140) (Fig. 2). The surveys were conducted for 2 years, from September 2017 to 2019.

The adult collections were performed from 08:00 to 15:00 h, and collections lasted for 15 min at each selected location. Mosquito collections from the selected houses/bedrooms/outdoor places were performed according to the WHO guidelines using a Prokopack aspirator. The collection began at the entry point and continued in a clockwise direction. The collection was made in four sessions, ensuring the minimum disturbance using four collection cups separately. First, the collections were made targeting resting places that were closer to the approximate height of < 1 m from the ground level such as underneath furniture, the bottom part of hangings, etc., at indoor resting places and small shrubs, grass, outdoor walls and other possible resting surfaces at outdoor sites. Later, the resting site at the next level of heights (1–2 m, 2–3 m and > 3 m) were reached by approximation of height from the floor. A total of 1482 premises were surveyed during the surveillance period. All collected mosquitoes were identified to the species level by referring to the morphological taxonomic key for *Aedes* mosquitoes [25].

### Detection of host-seeking hours of adult mosquitoes

Human-baited double-net traps were used to determine the host-seeking time of *Aedes* mosquitoes using the standard traps according to the specified guidelines [26], and the trial was repeated three times. A trained Field Assistant volunteered, and written informed consent was obtained from the participants. The participants rested on a metal-framed bed net (20 × 200 × 70 cm). They were fully protected from mosquitoes by an internal polyester bed net (97 × 200 × 100 cm, mesh size 1.5 mm), which was not treated with any insecticide. It was hung over the bed to the ground. A larger untreated bed net (100 × 250  × 150 cm, mesh size 1.5 mm), also not treated with insecticide, was hung over the smaller net. It was raised 30 cm above the ground. The mosquitoes caught in the ± 20 cm gap between the two nets were collected using a mouth aspirator every 10 min per hour. Mosquito collections were continued from 05:00 to 19:00 h. Atmospheric temperature and relative humidity were recorded hourly with mosquito collections. The mosquitoes collected each hour were transferred into different paper cups covered with a nylon mesh (1.5 mm). The mosquitoes collected from genus *Aedes* were authenticated to species level using standard morphological keys [25].

### Data analysis

The prevalence of *Ae. aegypti* and *Ae. albopictus* immature stages (larvae and pupae) in different breeding sites were used to calculate the container index (CI), which refers to the percentage of positive containers over the total number of water-holding containers inspected [22]. All recorded data were analysed using Statistical Package for Social Sciences (SPSS), version 21. The difference in the distribution of *Ae. aegypti* and *Ae. albopictus* at different environmental setups was analysed using the univariate general linear model (GLM) followed by Tukey’s HSD (honest significant difference) at a 5% significance level. The variation in the oviposition site preferences in terms of CI for both species was analysed by multivariate GLM at a 5% significance level. The difference between exophilic and endophilic resting behaviour of adult mosquitoes was analysed using the chi-squared test of independence at a 95% of confidence level. The mean number of adult female mosquitoes was calculated. Pearson’s correlation was used to analyse the relationship between mean numbers of female mosquitoes per hour, with mean atmospheric temperature and relative humidity at 5% significant intervals.

## Results

### Oviposition preferences of *Aedes* mosquitoes at different environmental setup

**Fig. 3:**
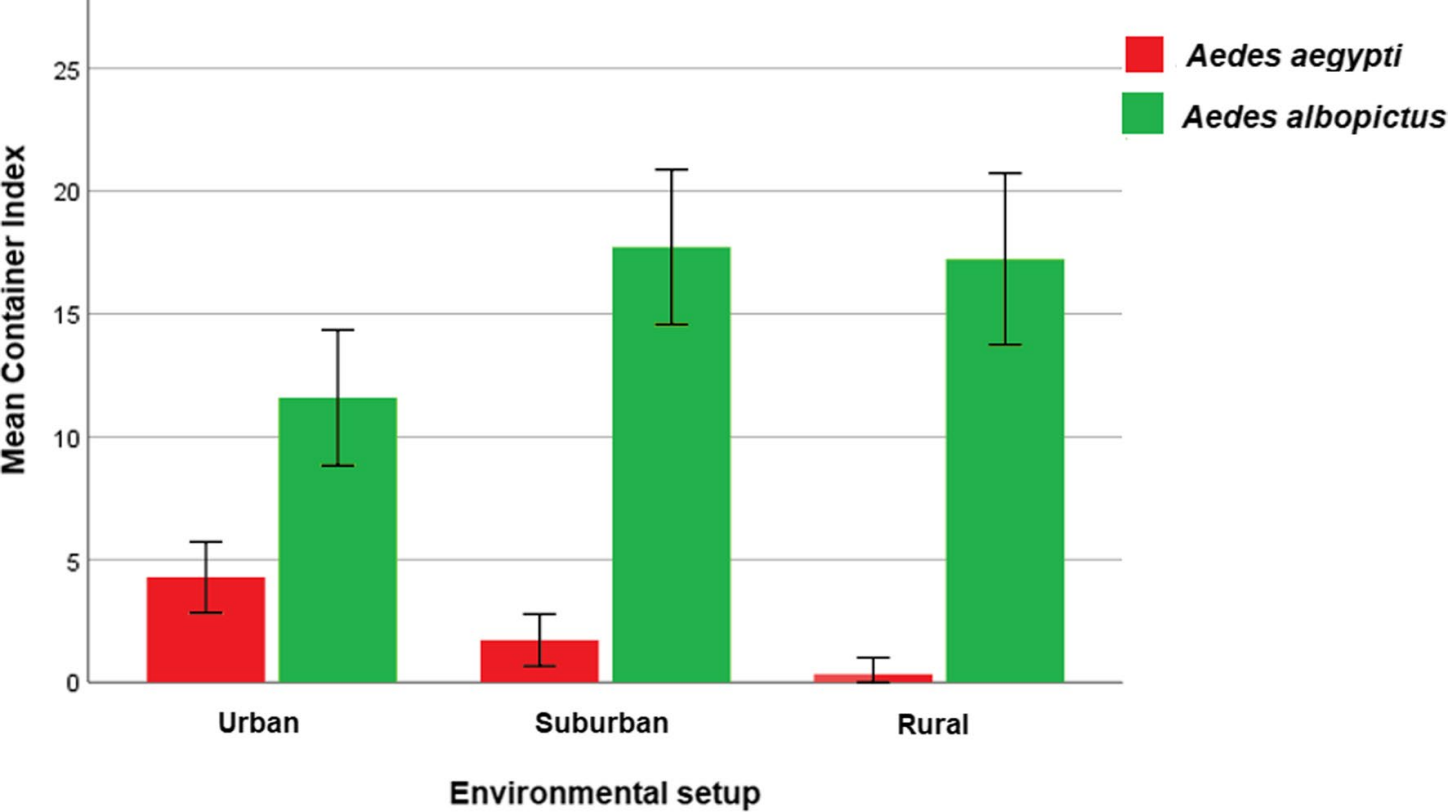
Distribution of *Ae. aegypti* and *Ae. albopictus* in terms of mean container index at different environmental settings representing urban, suburban and rural areas

A total of 19,829 potential breeding sites (water-holding containers/wet containers; *n* = 10,032, dry potential containers; *n* = 9797), both temporary and permanent, were examined at 13,563 premises. Of them, 18.5% (*n* = 1856) of water-holding containers were positive for *Aedes* larvae. *Aedes albopictus* was observed from all spatial setups (rural, suburban and urban), denoted as the leading species over *Ae. aegypti*. However, urban areas were more conducive for *Ae. aegypti* breeding. The container index (CI) for *Ae. albopictus* was highest in rural areas, followed by suburban setup. *Aedes aegypti* was identified mainly from urban and suburban setups (Table 1). According to GLM, the distribution of *Ae. aegypti* and *Ae. albopictus* was statistically significant at species level (*df* = 1; *F* = 137.134; *P* < 0.05) and spatially in selected areas in the Gampaha district (*df* = 2; *F* = 8.125; *P* < 0.05). Figure 3 represents the distribution of *Ae. aegypti* and *Ae. albopictus* in terms of mean container index at different environmental settings representing urban, suburban and rural areas.

**Table 1:** Container index of *Aedes* mosquitoes encountered at different study setups in Gampaha District, Sri Lanka

Breeding habitat types	Container index							
	*Aedes aegypti*				*Aedes albopictus*			
	Urban	Suburban	Rural	Total	Urban	Suburban	Rural	Total
Water storage barrels	7.1	3.1	**1.4**	3.5	11.2	13.2	18.4	14.7
Water storage cement tanks	–	5.1	–	1.7	8.0	7.7	21.6	13.9
Water storage other	1.6	0.5	–	0.5	1.6	10.6	12.9	10.0
Concrete slab	2.9	**14.6**	–	5.4	**20.0**	4.2	15.4	12.8
Gutters	–	–	–	–	9.4	6.3	19.2	11.3
Tyres	14.0	8.8	–	**6.6**	14.0	31.6	31.7	27.9
Ornamentals	5.7	1.8	0.4	2.1	13.6	18.6	23.6	19.5
Natural	2.4	–	–	0.2	13.1	26.5	19	22.4
Ponds	–	–	–	–	–	25.0	12	6.5
Wells	–	–	–	–	–	2.9	3.3	1.0
Tube wells	5.0	–	–	3.9	16.8	11.1	18.5	16.8
AC/refrigerator	4.5	0.6	–	1.9	3.1	5.0	19	8.7
Temporary removals	6.8	0.5	0.6	2.5	8.6	20.1	19.9	16.5
Covering items/polythene	4.8	2.3	–	2.1	18.0	18.8	31.7	22.7
Discarded degradable	0.7	–	–	0.3	13.0	26.5	17.8	18.3
Discarded reusable items	9.8	0.4	–	1.9	14.1	25	14.5	19.3
Discarded non-reusable items	5.3	–	–	1.9	12.5	20.7	18.2	17.0
Pet feeding cups	2.6	–	–	1.1	4.5	11.3	17.0	9.1
Non-used cisterns/commode	**14.3**	5.9	–	6.0	14.3	**40.8**	**32.1**	**36.8**
OtherA	–	–	–	–	7.7	11.5	8.3	10.7
Total	4.2 (125/2948)	1.3 (51/4033)	0.2 (5/3051)	1.8 (181/10032)	9.5 (281/2948)	19.4 (785/4033)	20.0 (609/3051)	16.7 (1675/10038)

Container index was calculated as number of positive containers for *Aedes* spp./total number of water-holding containers inspected × 100

The bolded values indicate the highest CI observed for *Aedes aegypti* and *Aedes albopictus* at each environmental setup and overall CI for each species recorded from all environmental setup

### Breeding site preference of *Aedes* mosquitoes

A total of 20 breeding site categories for *Aedes* mosquitoes were identified. *Aedes aegypti* was recorded in 16 larval habitat types, and *Ae. albopictus* was recorded from all categories. The statistics of GLM showed that the percentage positivity of *Aedes* mosquitoes varied significantly, among MOH areas (*Ae. aegypti*; *df* = 5; *F* = 47.9; *P* < 0.05, *Ae. albopictus*; *df* = 5; *F* = 28.261; *P* < 0.05) and breeding site categories (*Ae.aegypti*; *df* = 19; *F* = 48.1; *P* < 0.05, *Ae. albopictus: df* = 19; *F* = 20.171, *P* < 0.05).

**Fig. 4:**
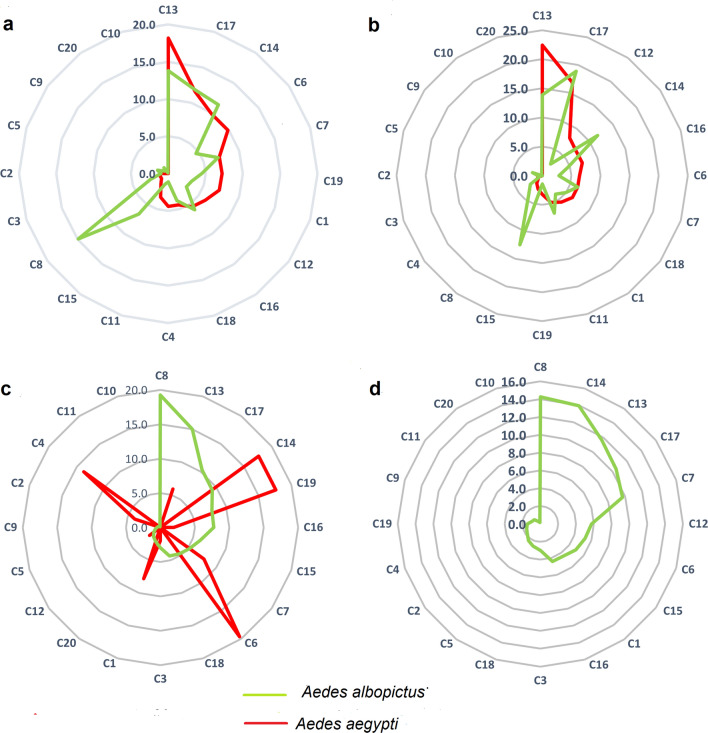
Percentage occurrence of each positive breeding site out of the total number of positive breeding sites inspected. **a** Study areas representing all environmental settings, **b** urban environmental setting, **c** suburban environmental setting and **d** rural environmental setting. C1, water storage barrels; C2, water storage cement tank; C3, water storage other; C4, concrete slabs; C5, gutters; C6, tyres; C7, ornamentals; C8, natural; C9, ponds; C10, wells; C11, tube wells; C12, AC/refrigerator trays; C13, temporary removals; C14, covering items/polythene; C15, discarded degradable items; C16, discarded reusable items; C17, discarded non-degradable items; C18, pet feeding cups; C19, non-use cisterns and commode; C20, other

The breeding of *Ae*. *aegypti* was more conducive in temporary removals (19.0%; *n* = 34), discarded non-reusable items (12.0%; *n* = 21), tyres (10.1%; *n* = 18) and covering items (10.1%; *n* = 18). *Aedes albopictus* mosquitoes were predominant in natural breeding places (14.7%; *n* = 246), temporary removals (13.6%; *n* = 227), discarded non-reusable items (12.0%; *n* = 198), covering items/polythene (11.5%; *n* = 192) and ornamentals (7.10%; *n* = 119) (Fig. 4). A pictorial collection of breeding sites identified in the field surveys is included as supplementary material (Additional file 1: Fig. S1).

Although temporary removal items and discarded items were recognized as the most frequent breeding sites with higher percentage occurrence for *Ae. aegypti*, tyres (6.6%), non-used cisterns/commode (6%) and concrete slabs (5.4%) were identified as the preferable breeding places for *Ae. aegypti* indicating higher container indices. The highest container type indices for *Ae. albopictus* were also reported for non-used cisterns/commode (36.8%), tyres (27.9%) and covering items/polythene (22.7%) (Table 1).

### Resting preferences of *Ae. aegypti* and *Ae. albopictus* adult mosquitoes

A total of 1834 adult mosquitoes were collected from indoor and outdoor resting locations inspected. Of the collections, 80.8% (*n* = 1482) represented by females. The majority of the collection comprised of *Ae. albopictus* (54.5%; *n* = 999). *Aedes aegypti* adult mosquitoes were detected mostly from indoor resting places, and outdoor resting places were more prominent for *Ae. albopictus* (Table 2). The chi-square analysis showed that the observed difference of two vectors for indoor and outdoor resting preferences was statistically significant at 95% confidential intervals (*χ*^2^ = 1025; *df* = 1; *P* < 0.001). In terms of resting places, *Ae. aegypti* was mostly found in resting places such as bedrooms (18.4%; *n* = 337) followed by living rooms (11.3%; *n* = 207) and kitchens (4.7%; *n* = 86). Outdoor resting places were least preferred by *Ae. aegypti*. On the other hand, *Ae. albopictus* was conducive to rest on the vegetation (25.1%; *n* = 460) in peri-domestic and house backyard (Table 2).

**Table 2:** Frequency and relative prevalence of adult mosquitoes collected at different indoor and outdoor resting places

Species and gender	Indoor							Outdoor				Total collected
	Bathroom (*n* = 1236)	Bedroom (*n* = 1186)	Dining room (*n* = 1186)	Kitchen (*n* = 1230)	Living room (*n* = 1085)	Store room (*n* = 1184)	Total	Outside front (1236)	Outside back (*n* = 1236)	Vegetation (*n* = 1236)	Total	
*Ae. aegypti*												
Female	26 (3.7%)	304 (43.1%)	78 (11.1%)	76 (10.8%)	173 (24.5%)	16 (2.3%)	673 (95.5%)	11 (1.6%)	7 (1.0%)	14 (2.0%)	32 (4.5%)	705 (38.4%)
Male	3 (2.3%)	33 (25.4%)	5 (3.8%)	10 (7.7%)	34 (26.2%)	–	85 (65.4%)	14 (10.8%)	23 (17.7%)	8 (6.2%)	45 (34.6%)	130 (7.1%)
Total	29 (3.5%)	337 (40.4%)	83 (9.9%)	86 (10.3%)	207 (24.8%)	16 (1.9%)	758 (90.8%)	25 (3.0%)	30 (3.6%)	22 (2.6%)	77 (9.2%)	835 (45.5%)
*Ae. albopictus*												
Female	10 (1.3%)	40 (5.1%)	14 (1.8%)	29 (3.7%)	47 (6.0%)	–	140 (18.0%)	114 (14.7%)	205 (26.4%)	318 (40.9%)	637 (82.0%)	777 (42.3%)
Male	1 (0.5%)	2 (0.9%)	6 (2.7%)	–	8 (3.6%)	–	17 (7.7%)	35 (15.8%)	28 (12.6%)	142 (64.0%)	205 (92.3%)	222 (12.1%)
Total	11 (1.1%)	42 (4.2%)	20 (2.0%)	29 (2.9%)	55 (5.5%)	–	157 (15.7%)	149 (14.9%)	233 (23.3%)	460 (46.0%)	842 (84.3%)	999 (54.5%)
Total collected	40 (2.2%)	379 (20.7%)	103 (5.6%)	115 (6.3%)	262 (14.3%)	16 (0.9%)	915 (49.9%)	174 (9.5%)	263 (14.3%)	482 (26.3%)	919 (50.1%)	1834 (100%)

Values in parentheses refer to the frequencies of the number of adult mosquitoes collected for *Ae. aegypti* and *Ae. albopictus* (relative abundance based on total adult mosquitoes collected), *n* represents the number of resting places inspected

*Aedes aegypti* female mosquitoes rested on cloth hangings and curtains, followed by furniture (13.2%; *n* = 243). Outside walls were the least preferable resting surface for *Ae. aegypti* (1.0%; *n* = 21). The highest percentages of *Ae. albopictus* mosquitoes were resting on vegetation in outdoor dwellings (25.1%; *n* = 460). According to the chi-square analysis, the preferences in resting surface among two mosquito species were statistically significant (*χ*^2^ = 1049; *df* = 1; *P* < 0.001).

It was interesting to note that the highest percentage of *Aedes* mosquitoes were found resting on wooden surfaces in both indoor and outdoor sites (44.2%; *n* = 811), clothes/curtains (22.8%; *n* = 418) and cement surfaces (22.6%; *n* = 415). Among two species, *Ae. aegypti* preferred cloths and hangings (16.8%; *n* = 308). *Aedes albopictus* mosquitoes rested on wooden surfaces (29.4%; *n* = 540). Furthermore, it was observed that the majority (45.7%; *n* = 839) of the resting population of *Aedes* mosquitoes was identified on the resting places closer to the ground level (< 1 m) followed by surfaces at 1–2 m height and 2–3 m (Table 3). Only 3.7% of the places > 3 m of height were identified as the least preferred resting sites for *Aedes* adult mosquitoes. However, no statistical differences were observed in terms of resting height with two species or gender of the mosquito species.

**Table 3:** Frequency and relative percentage of adult mosquitoes collected at different resting heights

Resting height (m)	*Aedes aegypti*			*Aedes albopictus*			Total
	Female	Male	Total	Female	Male	Total	
< 1	389 (55.2%)	51 (39.2%)	440 (52.7%)	320 (41.2%)	79 (35.6%)	399 (39.9%)	839 (45.7%)
1–2	214 (30.4%)	71 (54.6%)	285 (34.1%)	405 (52.1%)	111 (50.0%)	516 (51.7%)	801(43.7%)
2–3	73 (10.3%)	4 (3.1%)	77 (9.2%)	37 (4.8%)	12 (5.4%)	49 (4.9%)	126(6.9%)
> 3	29 (4.1%)	4 (3.1%)	33 (4.0%)	15 (1.9%)	20 (9.0%)	35 (3.5%)	68 (3.7%)

Values in parentheses refer to the frequencies of the number of adult mosquitoes collected for *Ae. aegypti* and *Ae. albopictus*

### Biting behaviours and peak biting hours of *Aedes* mosquitoes

A total of six mosquito species were recorded from human-baited double-net traps, namely, *Ae. aegypti*, *Ae. albopictus*, *Culex gelidus*, *Culex quinquefasciatus*, *Mansonia uniformus* and *Armigeres subalbatus*. However, 73.2% of the collection (*n* = 161/273) represented the mosquitoes under genus *Aedes* (*Ae. aegypti*—25.6%, *n* = 70; *Ae. albopictus*—33.3%; *n* = 91). Both *Ae. aegypti* and *Ae. albopictus* exhibited a typical diurnal pattern with two host-seeking and biting activity peaks, one in the morning and one in the afternoon.

**Fig. 5:**
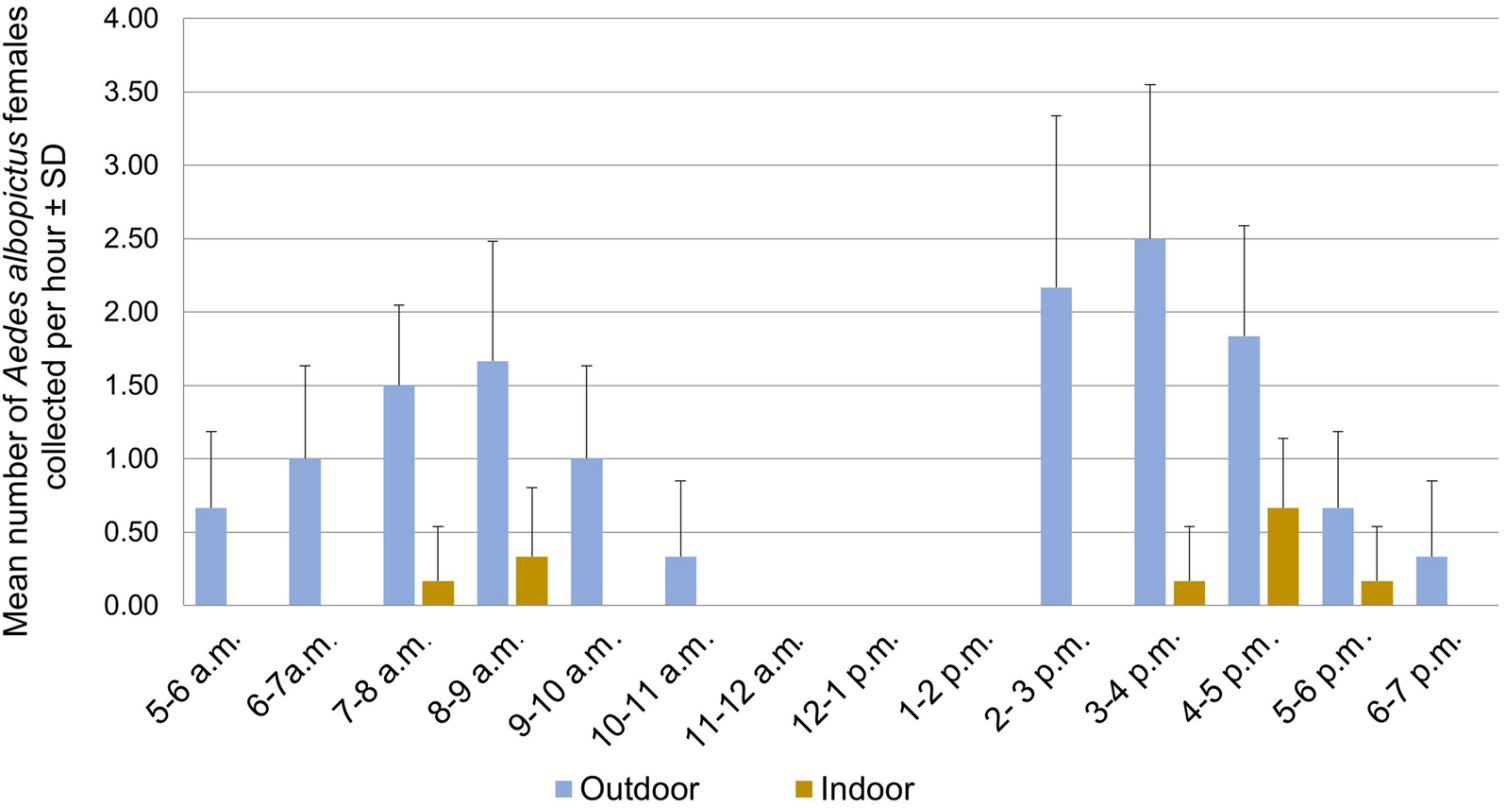
Indoor and outdoor host-seeking activity at different time periods of the day by *Ae. albopictus* adult females

**Fig. 6:**
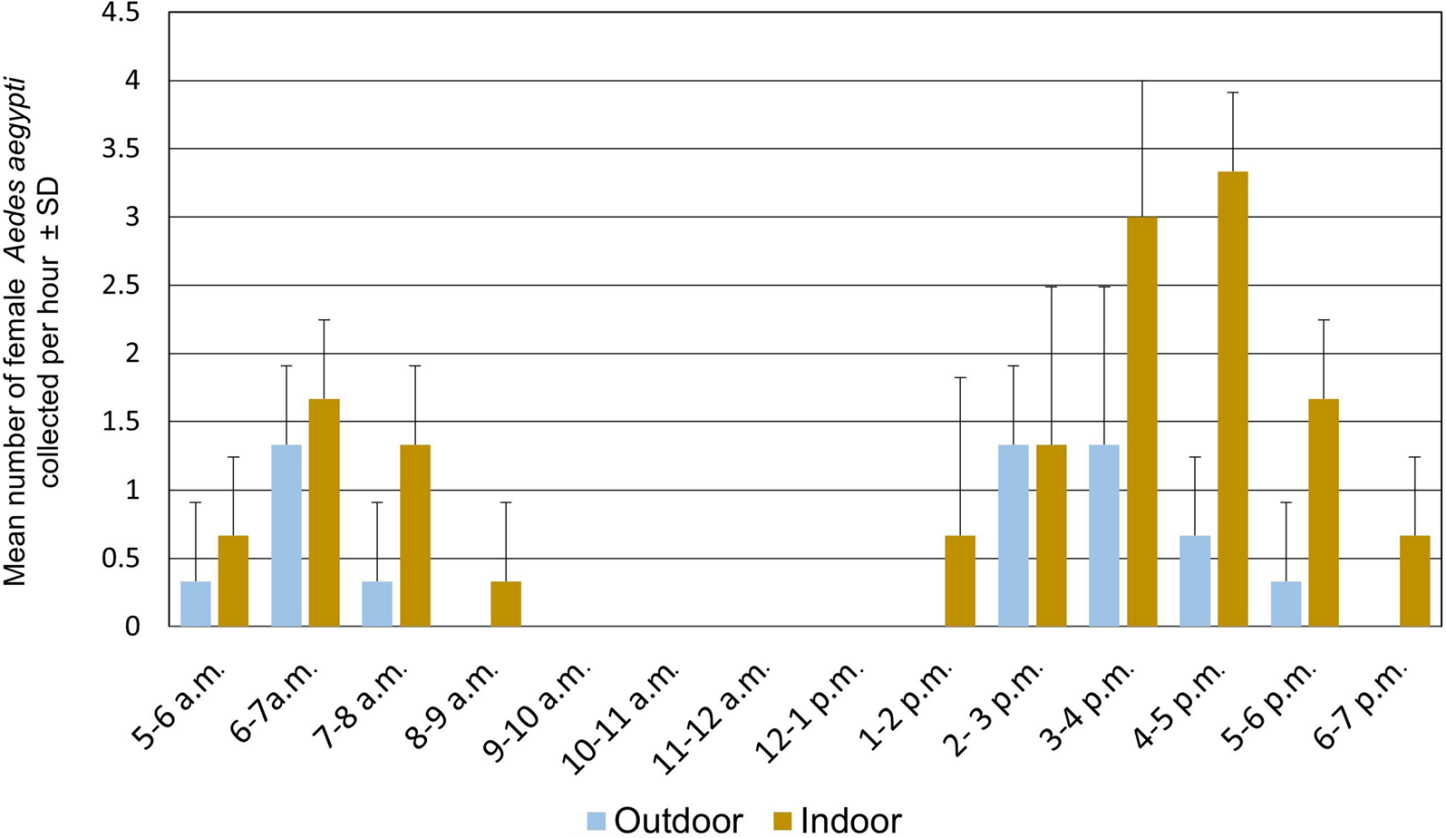
Indoor and outdoor host-seeking activity at different time periods of the day by *Ae. aegypti* adult females

The host-seeking cycle of *Ae. albopictus* was bimodal with two equally dominated peaks; morning peak occurred at 05:00–11:00 h and afternoon peak between 14:00 and 19:00 h. *Aedes aegypti* also demonstrated the same bimodal host-seeking cycle. A minor peak occurred between 05:00 and 09:00 h in the morning and a major peak between 13:00 and 19:00 h in the afternoon. It is interesting to note that *Ae albopictus* is more active outdoors in the morning and afternoon (Fig. 5). In contrast, *Ae aegypti* is more active indoors in the afternoon peak (Fig. 6).

The host-seeking activity of *Ae albopictus* is prominent in the early afternoon from 2 to 5 p.m. for a broader period outdoors. *Aedes aegypti* was active in a narrower period from 3 to 5 p.m. late evening (Figs. 5 and 6). Results indicate that both mosquito species shift their host-seeking locations only from outdoors to indoors but not vice versa in both periods. There was no significant correlation between the mean numbers of female mosquitoes caught with mean atmospheric temperature (*r* = 0.1; 30.7 ℃ ± 1.7 ℃) or relative humidity (*r* = − 0.3; 72.5% ± 5.4) according to the Pearson’s correlation.

## Discussion

The results of the present study document the oviposition/breeding site preferences, resting, biting and peak biting hours of *Ae. aegypti* and *Ae. albopictus* mosquitoes in Sri Lanka. Identifying vector distribution, density and percentage abundance, breeding habitats and behavioural aspects plays a critical role in evaluating current vector control strategies and further facilitating localized interventions that are specific to a particular region [27]. Therefore, the present investigation adds some fundamental information on the bionomics of dengue vector mosquitoes which can aid the designing of appropriate vector control interventions.

The current study’s findings showed that urban areas with higher dengue incidence were more conducive to *Ae. aegypti* breeding than suburban and rural areas. However, the present study also identified *Ae. albopictus* as the most abundant vector in all spatial setups in Gampaha District, including rural, suburban and urban areas. The study’s findings are supported by a few similar studies on the distribution and prevalence of these vectors in Sri Lanka [28, 29].

Reports from different countries, including Africa [30], Southeast Asia [31] and Australia [32], have described the elusive resting habits of *Ae. aegypti* [33]. However, some studies conducted in Panama [34], Costa Rica [35], Dominican Republic [36], Puerto Rico [37] and Mexico [38] evidenced the seclusive resting behaviour of *Ae. aegypti* within houses [18]. Furthermore, some investigations have indicated the presence of dengue infected *Ae. aegypti* in and around houses [39, 40]. Therefore, it is shown that houses provide a suitable environment for human-vector contact, dengue transmission and suitable resting sites for adult mosquitoes [18]. In addition, identifying the resting behaviour and resting sites of vectors would be beneficial to targeting vector control interventions such such as indoor residual spraying (IRS) or intra-domiciliary spraying and insecticide-treated curtains as emergency control measures against *Ae. aegypti* [18, 41].

The present study revealed that resting behaviour varied between the two *Aedes* vector species, where *Ae. aegypti* adult mosquitoes had a highly endophilic nature, while *Ae. albopictus* demonstrated exophilic behaviour confirming previous findings [18, 34]. In this study *Ae. aegypti* was mainly found resting in bedrooms, living rooms and kitchens. However, *Ae. albopictus* was found resting especially in outdoor vegetation. Results of the present study are similar to those of previous studies conducted in Trinidad [18], Panama [34] and Mexico [39]. They suggest that a domestic environment with high human-vector contact, especially in urbanized areas, provides suitable breeding and resting sites for *Ae. aegypti* mosquitoes. On the other hand, *Ae. albopictus* is mainly found among the vegetation in rural and suburban areas.

A higher proportion of *Ae. aegypti* resting was observed in indoor, dark and hidden surfaces such as cloths and cloth hangings and under furniture in houses kept at 2 m elevation from the ground. Previous studies have emphasized that around 67% of the dengue-positive houses harboured *Ae. aegypti* mosquitoes [42]. Furthermore, some studies have shown the presence of dengue virus-positive mosquitoes inside houses even 27 days after the initial clinical diagnosis of a patient [43]. Therefore, the presence of these mosquitoes in houses could be directly associated with disease incidence and transmission in these areas. Hence, appropriate control interventions should be focused on suppressing the adult densities based on evidence on the resting behaviour of vector mosquitoes.

Some research studies have indicated that insecticide applications targeting *Ae. aegypti* mosquitoes may be more effective in controlling dengue transmission [18]. However, some studies have identified that thermal fogging has limited success in controlling dengue outbreak situations, giving the reason that insecticide droplets may not reach up to adult *Aedes* mosquitoes at rest mainly indoors in hidden localities [44–48]. Simultaneously, thermal space fogging is associated with socio-economic and environmental problems because of high cost, difficulty of application and harm to non-targeted beneficial insects such as bees [34, 46–48].

Several studies have highlighted that *Ae. aegypti* females remain indoors and stay longer at resting sites [18, 49]. This behaviour of *Ae. aegypti* leads to failure of conventional space spraying of insecticide targeting the suppression of adult mosquitoes since there is a minimal possibility of insecticide droplets reaching indoors, especially in bedrooms [18]. Therefore, it shows the resting behaviour of *Ae. aegypti* mosquitoes is primarily responsible for the success of vector control programmes [18, 34, 50]. Many countries, including Trinidad [51], Suriname [52], Dominican Republic [36] and Panama [34], reiterated that the location of these resting sites is also a primary reason for the failure of *Ae. aegypti* control. Therefore, health authorities should focus on more appropriate vector interventions based on biology and behavioural aspects of dengue vectors.

Considering the limitation in conventional ultra-low-volume (ULV) spraying or thermal space spraying, indoor residual spraying (IRS) could be more viable since this method allows the insecticide to contact the resting mosquito population on insecticide-treated surfaces. This may provide evidence on the efficacy of IRS programmes and indicates why some countries, including Sri Lanka, in the past controlled malaria [18], with no dengue and leishmaniasis, with considerable public health importance. Therefore, the health authorities should re-introduce the IRS programme to control disease vectors rationally, targeting one disease vector. It is also important to elaborate that all these activities should be initiated based on scientific evidence and surveillance-guided integrated vector control programmes.

Host-seeking and -biting activities were also examined in this study to determine the biting cycles of the two dengue vectors [53] according to the habitat settings in the study area to plan appropriate vector control and bite prevention [54]. Adding to this, Chaves et al. [53] reported that the difference in the feeding behaviour of vectors affects the transmission pattern of vector-borne diseases during different seasons around the globe [55]. The present study demonstrated that both dengue vectors showed diurnal feeding behaviour, where the host-seeking cycle of *Ae. albopictus* was bimodal with two equally dominant peaks: morning peak occurred at 05:00–11:00 h and afternoon peak between 14:00 and 19:00 h. Meanwhile, *Ae. aegypti* exhibited a typical diurnal pattern with a minor peak occurring between 05:00 and 09:00 h in the morning and a significant peak between 13:00 and 19:00 h in the afternoon. The results were consistent with similar studies done in Trinidad, West Indies [56], Cameroon [57] and Malaysia [54], and *Ae. aegypti* and *Ae. albopictus* adult females showed endophagic and exophagic feeding behaviour, respectively.

Breeding site preference and oviposition behaviour are important aspects of vector bionomics. The primary vector for dengue transmission, *Ae. aegypti*, originated in Africa but now has spread to other continents [7]. Mosquitoes in the genus *Aedes* show different choices for oviposition ranging from indoors to outdoors and natural vegetation to human-made containers [12]. *Aedes aegypti* is a highly domesticated urban mosquito that prefers to live with humans in their homes, feeds on humans and oviposits in human-made artificial containers [16].

The results of the present study corroborate that urban areas are favoured by *Ae. aegypti* over suburban or rural areas where females can obtain their blood meals, rest and oviposit more easily and frequently because of high human population density and higher availability of human-made breeding habitats. Since *Ae.aegypti* has a relatively short flight range [27], this drastic urbanization provides the ideal ecological conditions to support higher densities of *Ae.aegypti*, assuring close contact with crowded human populations, leading to dengue epidemic situations in urban areas [16, 58]. Another study conducted by Noordeen et al. [28] in the central province of Sri Lanka quoted similar findings.

*Aedes* mosquitoes breed in both artificial and natural breeding sites, which provide clear and unpolluted water indoors and outdoors [23, 59], at ground level and above in places such as roof gutters, overhead tanks, concrete slabs and receptacles on roofs and slab areas at any type of premises [23]. Furthermore, *Aedes* mosquitoes have been recorded in brackish and saline water in Sri Lanka [60, 61]. The present research reports refrigerator trays, ant traps, ornamental items including flower vases, abandoned fish tanks and ponds, water storage containers in toilets/bathrooms, and cisterns and squatting pans of non-used toilets as the major indoor breeding sites for *Aedes* mosquitoes. Discarded items including degradables (coconut shells, clay pots) and non-degradables (plastics, glass, metal), water storage tanks and barrels, temporary removal items (toys, buckets, cooking utensils, etc.), ponds, tube wells and shallow cement wells, used tyres, roof gutters, natural breeding places (leaf axils, bamboo stumps and tree holes), covering polythenes, concrete slabs and cement floors and pet feeders were identified as the major outdoor breeding sites.

Even though *Aedes* mosquitoes breed in different types of breeding sites, the productivity varies based on the living standards of the population, and it is seasonal and area-specific [19, 23, 62]. In an area with an irregular water supply and shortage of water, water storage tanks and barrels have been identified as the major breeding sites for dengue vectors [60]. Simultaneously, discarded containers are the most productive breeding sites in areas with poor solid waste management, especially in urban settings [61]. Discarded containers and domestic wells are the major breeding places for *Aedes* [29]. Findings of the present study indicated that *Ae. aegypti* mainly breed in discarded items, temporary removals and covering items in urban areas because of higher availability of breeding sites with poor solid waste management, agreeing with Louis et al. [62], while *Ae. albopictus* mosquitoes were more predominant in discarded items followed by natural breeding places. In the present investigation, since > 80% of the breeding places for *Ae. aegypti* larvae were human-made artificial containers, larvicidal-driven vector control interventions such as the application of temephos and biological controlling methods may not be successful. It was also noted that *Aedes* populations are maintained in indoor breeding sites during the dry season. Therefore, well-planned integrated vector management programmes should be initiated with the collaboration of all the multiple stakeholders, including local government bodies and other related ministries, targeting source reduction by improving garbage collection and disposal systems, law enforcement and, most importantly, enhancing the awareness of the community about the elimination of mosquito breeding places in a domestic environment to control ongoing dengue outbreaks in the district.

## Conclusions

*Aedes aegypti* prefers to rest indoors, especially in dark and shady areas such as cloth hangings and under furniture. *Aedes albopictus* showed mainly exophilic behaviour; they are mostly resting among vegetation in the peridomestic environment. The majority of the vector population was identified at indoor resting places. Therefore, the efficacy of using thermal space spraying of insecticides could be questionable. Hence, re-introduction of indoor residual spraying for vector control could be recommended. The biting time was 05:00–11:00 h in the morning and between 13:00 and 19:00 in the afternoon. Therefore, preventive measures and attention to minimizing vector biting are recommended during the peak biting hours. Discarded items, temporary removals and covering items were identified as critical breeding places for *Ae. aegypti*, and discarded items followed by natural breeding places were identified as the key breeding places for *Ae. albopictus* mosquitoes. This study shows the need for scientific-based surveillance, monitoring and decision making in vector control approaches in Sri Lanka. It could be advantageous to have a holistic vector control and surveillance programme targeting medically important disease vectors in Sri Lanka.

## Supplementary Information

**Additional file 1: Figure S1.** Breeding site categories observed during the field surveys: **a** water storage barrel, **b** ornamental item, **c** concrete slab, **d** tyre, **e** bamboo hole, **f** natural leaf axil, **g** covering polythene, **h** non-used commode /cistern, **i** temporary removals, **j** gutter, **k** discarded degradables/non-degradables, **l** pet feeders, **m** refrigerator tray.

## Data Availability

The study datasets are available from the corresponding author upon reasonable request.

## Abbreviations

GLM: Generalized linear model
IRS: Indoor residual spraying
MOH: Medical officer of health
ULV: Ultra-low volume
CI: Container index
